# Clinical relevance of thrombocytosis in primary care: a prospective cohort study of cancer incidence using English electronic medical records and cancer registry data

**DOI:** 10.3399/bjgp17X691109

**Published:** 2017-05-23

**Authors:** Sarah ER Bailey, Obioha C Ukoumunne, Elizabeth A Shephard, Willie Hamilton

**Affiliations:** University of Exeter Medical School, Exeter.; NIHR CLAHRC South West Peninsula, University of Exeter Medical School, Exeter.; University of Exeter Medical School, Exeter.; University of Exeter Medical School, Exeter.

**Keywords:** cancer, platelet count, positive predictive value, primary care, risk marker, thrombocytosis

## Abstract

**Background:**

Thrombocytosis (raised platelet count) is an emerging risk marker of cancer, but the association has not been fully explored in a primary care context.

**Aim:**

To examine the incidence of cancer in a cohort of patients with thrombocytosis, to determine how clinically useful this risk marker could be in predicting an underlying malignancy.

**Design and setting:**

A prospective cohort study using Clinical Practice Research Datalink data from 2000 to 2013.

**Method:**

The 1-year incidence of cancer was compared between two cohorts: 40 000 patients aged ≥40 years with a platelet count of >400 × 10^9^/L (thrombocytosis) and 10 000 matched patients with a normal platelet count. Sub-analyses examined the risk with change in platelet count, sex, age, and different cancer sites.

**Results:**

A total of 1098 out of 9435 males with thrombocytosis were diagnosed with cancer (11.6%; 95% confidence interval [CI] = 11.0 to 12.3), compared with 106 of 2599 males without thrombocytosis (4.1%; 95% CI = 3.4 to 4.9). A total of 1355 out of 21 826 females with thrombocytosis developed cancer (6.2%; 95% CI = 5.9 to 6.5), compared with 119 of 5370 females without (2.2%; 95% CI = 1.8 to 2.6). The risk of cancer increased to 18.1% (95% CI = 15.9 to 20.5) for males and 10.1% (95% CI = 9.0 to 11.3) for females, when a second raised platelet count was recorded within 6 months. Lung and colorectal cancer were more commonly diagnosed with thrombocytosis. One-third of patients with thrombocytosis and lung or colorectal cancer had no other symptoms indicative of malignancy.

**Conclusion:**

Thrombocytosis is a risk marker of cancer in adults; 11.6% and 6.2% cancer incidence in males and females, respectively, is worthy of further investigation for underlying malignancy. These figures well exceed the National Institute for Health and Care Excellence-mandated risk threshold of 3% risk to warrant referral for suspected cancer.

## INTRODUCTION

Cancer is one of the leading causes of death in developed countries, with over 163 000 cancer deaths in the UK in 2014.^1^ The commonest route to cancer diagnosis follows the development of symptoms, and definitive diagnosis by biopsy and access to specialist care often rely on a primary care physician to recognise the possibility of cancer. It is generally accepted that delay in symptomatic diagnosis is harmful.^2^^,^^3^ One feature of possible cancer has only recently been recognised to have diagnostic potential: a raised platelet count, or thrombocytosis.

Thrombocytosis is present in 1.5–2.2% of the primary care consulting population aged ≥40 years.^4^^–^^6^ A recent systematic review summarised the evidence from previous primary care studies.^7^ Four case-control studies reported associations between thrombocytosis in primary care and a future diagnosis of lung, renal, uterine, and colorectal cancer.^4^^–^^6^^,^^8^ A further four primary care studies found that thrombocytosis was not predictive of pancreatic, breast, ovarian, or oesophagogastric cancer.^9^^–^^13^ Revised UK national guidance for suspected cancer incorporates thrombocytosis in some of its recommendations for lung, oesophagogastric, and uterine cancers.^2^ However, no study has examined thrombocytosis in primary care for all cancers. This study aimed to address that gap.

## METHOD

### Data sources

This prospective cohort study used data from the UK Clinical Practice Research Datalink (CPRD) (www.cprd.co.uk), which holds anonymised electronic primary care records from roughly 8% of UK practices. All patients’ consultations, laboratory results, and referrals are available in numerical coded format. Linked data from the English Cancer Registry were obtained for all patients. The data linkage was carried out by a third party, the Health and Social Care Information Centre, now known as NHS Digital (https://digital.nhs.uk/).

### Patient sample

The cohort was a random sample of 50 000 patients who had had a primary care full blood count taken. Within this there were two sub-cohorts: patients with thrombocytosis and patients with a normal platelet count.

The thrombocytosis sub-cohort included 40 000 patients selected using four criteria:

- a platelet count of >400 × 10^9^/L, henceforth labelled ‘thrombocytosis’;
- no previous thrombocytosis;
- aged ≥40 years at the time of thrombocytosis; and
- the thrombocytosis event was recorded from 2000 to 2013 inclusive.

In the CPRD, only the year of birth is available (to protect anonymity), so an arbitrary date of birth of 1 July was assigned, leading to the exclusion of 25 patients nominally aged <40 years. Patients with a platelet count >1000 × 10^9^/L were excluded because some extreme values were biologically implausible; others potentially represented platelet malignancies. The index date was defined as the date of the patient’s first raised platelet count. Patients with cancer before the index date were excluded.

**How this fits in**

Thrombocytosis has previously been identified as a marker of some types of cancer (lung, colorectal, renal, and uterine), but not all sites have been studied, and the effects of age, sex, and change in platelet count over time have not been examined. This is the first study to estimate the overall risk of cancer in patients with thrombocytosis. The positive predictive value of thrombocytosis is 11.6% (95% confidence interval [CI] = 11.0 to 12.3) for males and 6.2% (95% CI = 5.9 to 6.5) for females. This rises to 18.1% (95% CI 15.9 to 20.5) for males and 10.1% (95% CI 9.0 to 11.3) for females if the patient has a second raised platelet count within 6 months. These figures are well above the 3% threshold used by the National Institute for Health and Care Excellence for suspected cancer investigation, and strongly suggest that cancer should be considered in patients showing thrombocytosis.

The second (comparison) sub-cohort was matched to a random selection of 10 000 patients from the thrombocytosis sub-cohort. Patients selected for blood testing are inherently different from the untested population and more likely to have an underlying condition. Therefore patients with a normal platelet count were a superior comparator to the general consulting population. Matching was by age (within 5 years), sex, and practice. Comparison patients also had a platelet count from 2000 to 2013 inclusive and their *first* platelet count in that time was in the ‘normal’ range of 150–400 × 10^9^/L, although they could have thrombocytosis subsequently. The index date for these patients was their platelet count nearest in time to the index date of their matched comparator. Because this index date was sometimes later than their first normal platelet count, 374 (3.7%) of these had thrombocytosis at their index date, and were excluded.

Power calculations indicated that 40 000 patients with thrombocytosis was sufficient to estimate an incidence of cancer of 5% with a margin of error no greater than 0.22%, using the upper bound of the 95% confidence interval (CI), and 10 000 patients was sufficient to estimate the same incidence of cancer with a margin of error no greater than 0.45% similarly.

### Outcome variables

All new cancer diagnoses (excluding non-melanoma skin cancer, which is commonly under-reported in the cancer registry) in the 2 years after the index date were identified by searching electronic patient records for any of 2134 cancer-related codes (available from the authors on request). This list of cancer codes has been used in several studies, and is collated into 22 common cancer sites, plus a miscellaneous group. The reasons for blood tests being ordered were unknown; however, the 12 symptoms most frequently recorded for patients in each cohort in the month before their blood test were reported (Appendix 1).

New cancer diagnoses were also extracted from the cancer registry records. Patients were included as having a cancer diagnosis if one was recorded in either the CPRD or the cancer registry. The earliest record of cancer in either the CPRD or the registry was assigned the diagnosis date. Stage at diagnosis was determined from registry data. Early-stage cancer was defined as either stage I or stage II, and late-stage cancer as either stage III or IV.

### Statistical methods and analysis

Analyses were stratified by sex throughout. The primary analysis reported the 1-year cancer incidence as a percentage (and 95% CIs) for patients with thrombocytosis and normal platelet count. For the thrombocytosis cohort this is equivalent to the positive predictive value (PPV) of thrombocytosis for cancer. The specific site of the cancer diagnosis was identified from the electronic records. Where more than one cancer was diagnosed, the first recorded cancer site was used. The time between first thrombocytosis and any cancer diagnosis was determined. The results were stratified by 10-year age bands. Where a second platelet count was recorded within 6 months of index date, the risk of cancer was estimated depending on whether the second value showed an increase or decrease in platelet count.

Patients diagnosed within 3 months of thrombocytosis may well have already entered diagnostic pathways, so potentially have less to benefit from thrombocytosis ‘triggering’ investigation. In contrast, expedition of diagnosis by >3 months may yield considerable survival benefit. Therefore, a secondary analysis examined two subgroups: those diagnosed 4–12 months, and those diagnosed 13–24 months after thrombocytosis.

Of the patients in the main cohort diagnosed with lung or colorectal cancer (the two most common cancer sites diagnosed), the study sought to examine the proportion with recorded symptoms that matched the contemporary National Institute for Health and Care Excellence (NICE) suspected cancer referral guidance,^2^ and the proportion with no cancer symptoms other than thrombocytosis.

All analyses were performed using Stata (version 13). The reporting of this study conforms to the STROBE statement.^14^

## RESULTS

**Figure 1.**
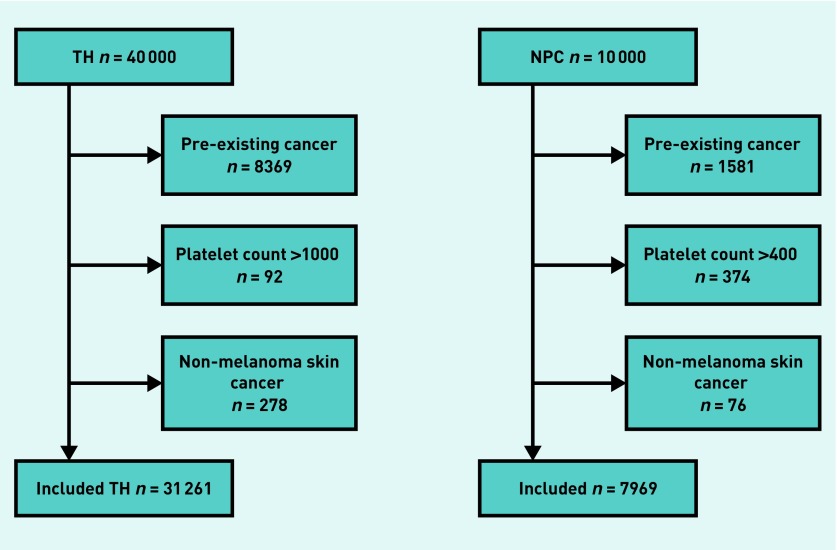
*Patient flow diagram to show the number of patients included in each cohort, and the number excluded for having pre-existing cancer, having a platelet count outside of the eligible range, or having non-melanoma skin cancer. NPC = normal platelet count. TH = thrombocytosis.*

After exclusions, the thrombocytosis cohort included 31 261 patients (Figure 1); the median age was 67.9 years (interquartile range [IQR] = 57.1 to 78.1 years), and 21 826 (69.8%) were female. The normal platelet count cohort included 7969 patients; the median age was 68.3 years (IQR = 58.1 to 78.5 years) and 5370 (67.4%) were female.

### Cancer diagnoses

**Figure 2a.**
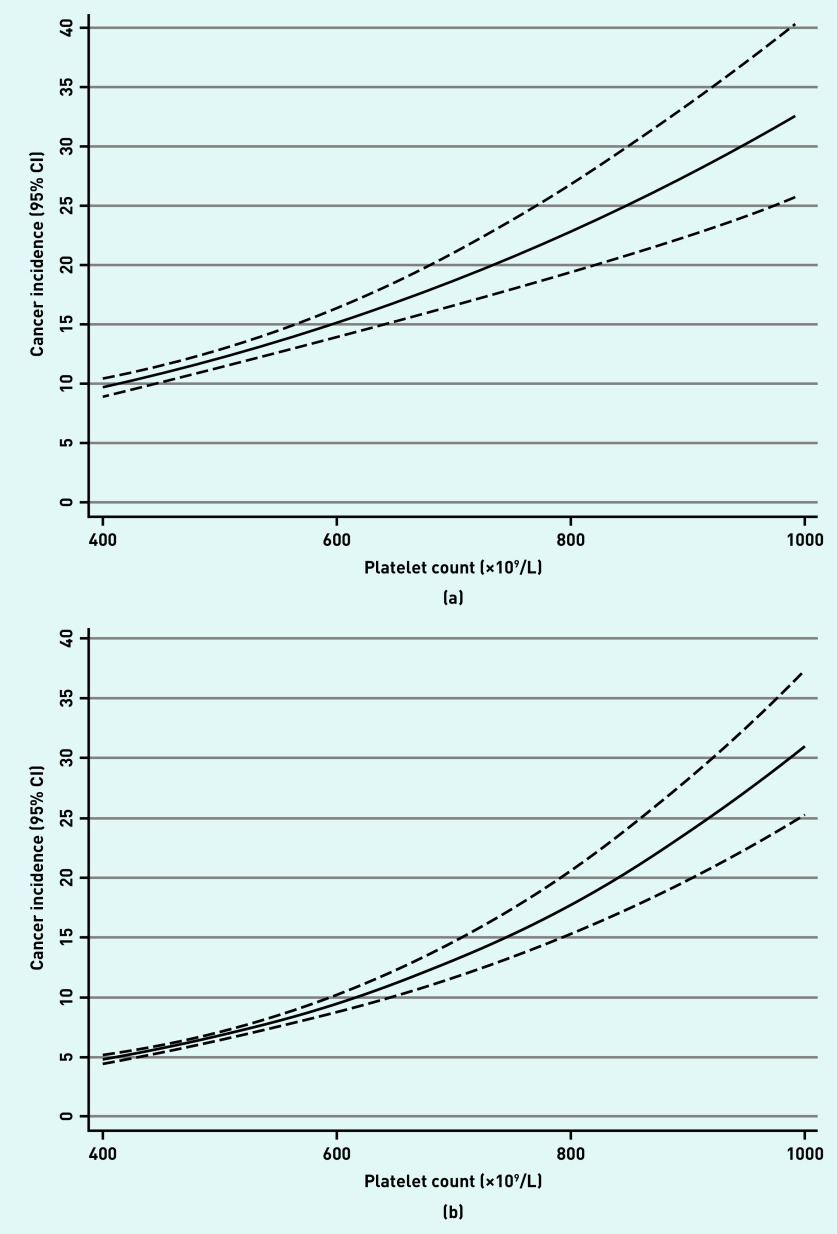
*1-year cancer incidence (solid line, with 95% CIs indicated by dashed lines) against platelet count as a continuous variable, for males aged ≥40 years. 2b. 1-year cancer incidence (solid line, with 95% CIs indicated by dashed lines) against platelet count as a continuous variable, for females aged ≥40 years. CI = confidence interval.*

**Figure 3a.**
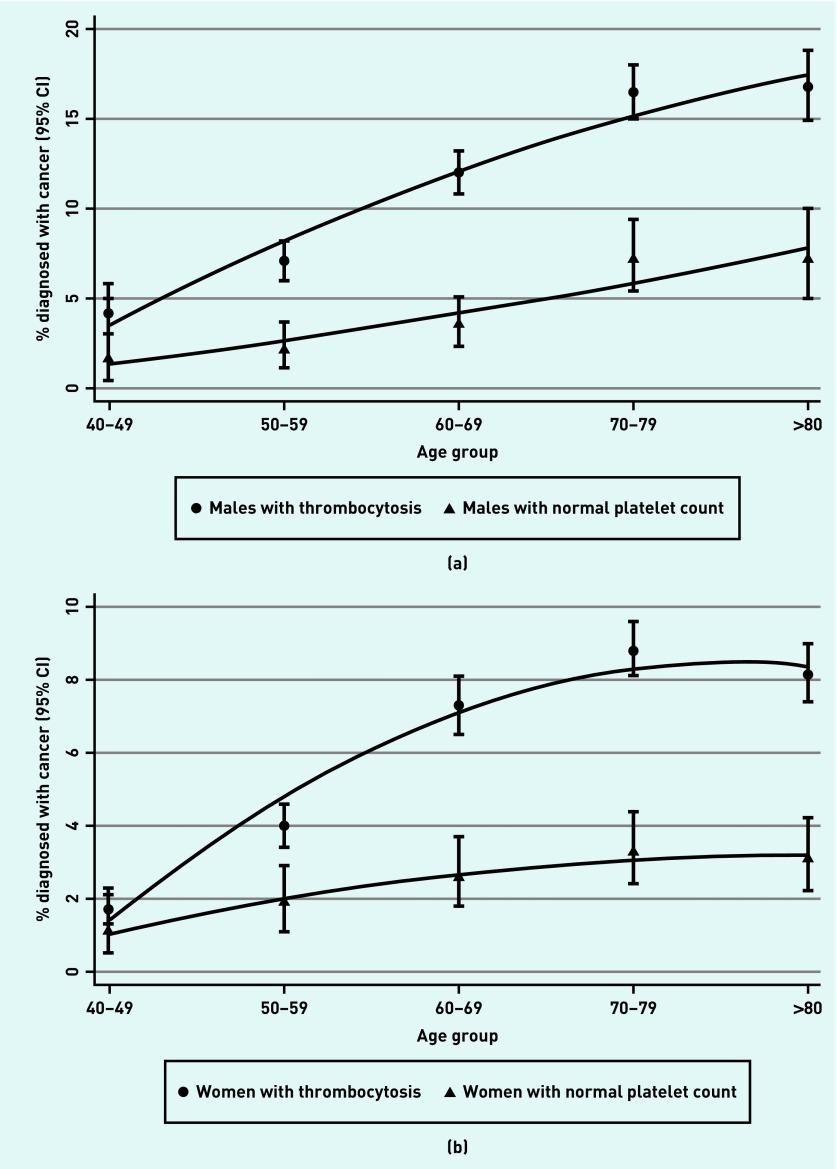
*Proportion of males diagnosed with cancer in the thrombocytosis and normal platelet count cohorts by age group, with 95% CI bars. 3b. Proportion of females diagnosed with cancer in the thrombocytosis and normal platelet count cohorts by age group, with 95% CI bars. CI = confidence interval.*

There were 1098 cancers diagnosed within 1 year of thrombocytosis in the sample of 9435 male patients, and 1355 in the sample of 21 826 female patients. This represents a 1-year cancer incidence of 11.6% (95% CI = 11.0 to 12.3) for males and 6.2% (95% CI = 5.9 to 6.5) for females; this is equivalent to the PPV of a raised platelet count. In patients with a normal platelet count there were 106 cancers diagnosed in males and 119 in females; a 1-year cancer incidence of 4.1% in males (95% CI = 3.4 to 4.9) and 2.2% in females (95% CI = 1.8 to 2.6) (Table 1). Approximately one-third of cancers in the thrombocytosis group were diagnosed at least 3 months after their index date (Table 1: 4–12 months). In the second year after index date, cancer incidence had returned to baseline levels (Table 1: 13–24 months). The risk of cancer increased with increasing platelet count (Figure 2). Patients with thrombocytosis were at consistently greater risk than those with normal platelet counts across all ages; the difference in risk between those with and without thrombocytosis increased from age 70 years. (Figure 3).

**Table 1.** The proportion of patients with thrombocytosis and normal platelet counts diagnosed with cancer, within 0–12, 4–12, and 13–24 months of platelet count index date

	**Thrombocytosis (*n*= 31 261)**				**Normal platelet count (*n*= 7969)**			
	
	**Males**		**Females**		**Males**		**Females**	
			
**Time from index date (months)**	***N***	**Cancers diagnosed, *n* PPV (95% CI)**	***N***	**Cancers diagnosed, *n* PPV (95% CI)**	***N***	**Cancers diagnosed, *n* % diagnosed (95% CI)**	***N***	**Cancers diagnosed, *n* % diagnosed (95% CI)**
0–12	9435	1098	21 826	1355	2599	106	5370	119
		11.6% (11.0 to 12.3)		6.2% (5.9 to 6.5)		4.1% (3.4 to 4.9)		2.2% (1.8 to 2.6)

4–12	8677^a^	340	20 974^a^	503	2544^a^	51	5321^a^	70
		3.9% (3.5 to 4.3)		2.4% (2.2 to 2.6)		2.0% (1.5 to 2.6)		1.3% (1.0 to 1.7)

13–24	8337^b^	224	20 471^b^	373	2493^b^	35	5251^b^	72
		2.7% (2.4 to 3.1)		1.8% (1.6 to 2.0)		1.4% (1.0 to 1.9)		1.4% (1.1 to 1.7)

a *Excluding patients with cancer diagnoses recorded within months 0–3.*

b *Excluding patients with cancer diagnoses recorded within months 0–12. PPV* = *positive predictive value.*

When patients had a second platelet count taken within 6 months of their first, the 1-year incidence of cancer was greatest in males whose next platelet count showed an increase in platelets or the same platelet count: 18.1% (95% CI = 15.9 to 25.2), or whose next platelet count decreased, but remained abnormally high: 19.1% (95% CI = 16.5 to 20.5). For females, these two groups were also at the greatest risk of cancer: 10.1% (95% CI = 9.0 to 11.3) and 7.3% (95% CI = 6.3 to 8.4), respectively.

Staging data were available for 1168 of the 2453 cancers diagnosed in the thrombocytosis cohort. Of these, 575 (49.2%) were early stage and 593 (50.8%) were late stage. In the normal platelet count cohort, staging data were available for 123 of the 225 cancers diagnosed. Of these, 77 (62.6%) were early stage and 46 (37.4%) were late stage.

There were 2440 diagnoses recorded in the CPRD data (from either cohort) and 3078 in the cancer registry data: 2136 of the 2440 CPRD-recorded diagnoses were validated by the cancer registry data (positive predictive value of 87.5%); 2136 of 3078 cancer registry-recorded diagnoses were captured by the CPRD (sensitivity of 69.4%).

### Specific cancer sites

**Figure 4a.**
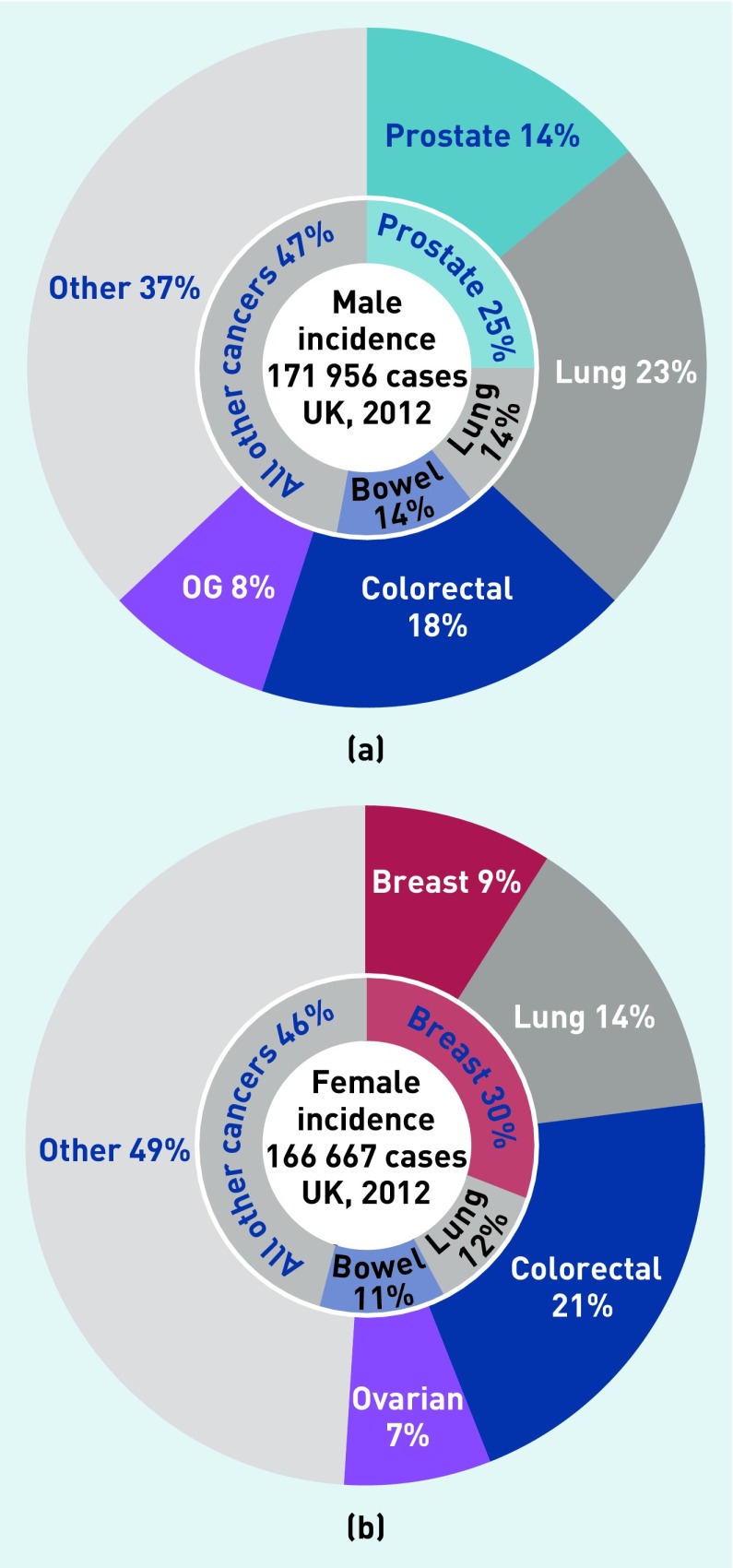
*The most commonly diagnosed cancer types in males with thrombocytosis, compared with males in the general population. Outer ring shows the cancers diagnosed in males with thrombocytosis. Inner ring shows incidence of cancer types in the general population. 4b. Commonly diagnosed cancer types in females with thrombocytosis, compared with females in the general population. Outer ring shows cancers diagnosed in females with thrombocytosis. Inner ring shows incidence of cancer types in the general population. OG = oesophagogastric.*

Lung and colorectal cancers were the most commonly diagnosed cancers in the thrombocytosis cohort. Lung and colorectal cancer were much more commonly diagnosed in patients with thrombocytosis than in the general population, and breast and prostate cancer were much less commonly diagnosed (Figure 4).

### Symptoms accompanying thrombocytosis

The 12 most common symptoms reported in each group are shown in Appendix 1. There was little difference between the two cohorts in symptom reporting in the month before their blood test. Data on the different cancers is available from the authors on request. Of the 31 261 patients with thrombocytosis, 573 were diagnosed with lung cancer; 195 (35.7%) had no symptoms warranting urgent investigation as per NICE guidance, other than thrombocytosis. Similarly, 627 of the 31 261 patients with thrombocytosis were diagnosed with colorectal cancer; 206 (32.9%) had no symptoms in the year before diagnosis warranting urgent investigation for cancer. The median number of days between thrombocytosis and diagnosis date was 50 for lung cancer (IQR = 18 to 126) and 67 for colorectal cancer (IQR = 27 to 174). Thus, in at least one-third of all lung and colorectal cancer patients, thrombocytosis could potentially have triggered investigation in a patient hitherto not meeting current recommendations for referral.

## DISCUSSION

### Summary

This large-scale cohort study is the first from primary care to report the overall risk of cancer in patients with thrombocytosis, compared with those with normal platelet counts. Males with thrombocytosis had an 11.6% incidence of cancer in the following year, and females had an incidence of 6.2%: this compares with 4.1% of males (95% CI = 3.4 to 4.9) and 2.2% of females (95% CI = 1.8 to 2.6) (Table 1) with normal platelet counts. The incidence of cancer rose with age and with a higher platelet count, and at least one-third of patients with lung and colorectal cancer with pre-diagnosis thrombocytosis had no other symptoms indicative of malignancy.

### Strengths and limitations

A key strength of this study is its size and setting in primary care, where the initial suspicion of possible cancer generally occurs.

The inclusion of English Cancer Registry data is also a strength. These results rely on the accuracy of data in the CPRD. Recent publications have found fairly high levels of concordance between the CPRD and cancer registries: 83–92%.^15^^,^^16^ The percentage of total cancers identifiable from the CPRD alone in this study (69.4%) probably reflects the dataset ranging from 2000 to 2013, and the inclusion of any cancer type (except non-melanoma skin cancer). Cancer recording in the CPRD has improved in recent years, and the inclusion of any cancer type (except non-melanoma skin cancer) the likely explanation for the superior figures above.

Blood test results are electronically transmitted to patient records, reducing the likelihood of misclassification bias, although a small number of participants may have had previous thrombocytosis before joining their current CPRD practice. Registry staging data were incomplete; therefore, it is only possible to conclude that at least half of cancers diagnosed in patients with thrombocytosis were early stage and most likely to benefit from earlier diagnosis in terms of survival, though this proportion may be higher. This result should be interpreted with caution, and further analysis was not attempted; the large amount of missing data suggests a bias in those patients for whom staging data are available.

The symptoms preceding blood testing were reported by small, and similar, proportions of each cohort. Additionally, all are low-risk symptoms, which would not generally be expected to prompt urgent cancer investigation.^2^ It is unlikely that many of the blood tests carried out in the thrombocytosis group were triggered by a specific suspicion of cancer, as the symptoms were so similar between the groups.

In primary care, blood tests are ordered for a wide range of reasons, rarely to test for thrombocytosis specifically; approximately one-quarter of the UK adult primary care population have a full blood count in any one year.^17^ Patients who have had blood tests are expected to have more ill health than those who are not tested, thus a comparison cohort of patients with a normal platelet count was included, rather than a group with no testing at all. This should reduce (or eliminate) selection bias from patients being chosen for blood testing. This study does not report the value of *measuring* the platelet count in possible cancer; instead it reports the clinical usefulness of a thrombocytosis, which may be, and often is, a serendipitous finding in primary care. This is a subtle but real distinction.

### Comparison with existing literature

Few studies have investigated the association between thrombocytosis and cancer in primary care. The results of this study agree with the four earlier single cancer site studies,^4^^–^^6^^,^^8^ three of which used the CPRD as a data source, although these three had different study designs from the current study and used different patients. Four further studies found no association between thrombocytosis and a later cancer diagnosis.^9^^–^^11^^,^^13^ These studies were examined in a recent systematic review.^7^

In secondary care, a study of patients with chest X-ray abnormalities suggesting possible lung cancer examined pre-diagnostic platelet counts, finding thrombocytosis in 57% of patients with lung cancer and in 8% of those with benign lung disease.^18^ In primary care, the percentage of patients with lung cancer who had thrombocytosis is 14%, although this lower figure may arise from testing having been performed earlier in the diagnostic pathway when fewer patients are likely to have an underlying malignancy.^5^ These two studies,^5^^,^^19^ plus results from the current study, suggest that in some patients with lung cancer the platelet count rises progressively in the months before diagnosis. This is supported by the finding that thrombocytosis was more common in patients with advanced disease.^18^

There are three main theories underlying the association between platelets and cancer. In patients with ‘pre-existing’ thrombocytosis, tumour development may be augmented by platelet-released proangiogenic cytokines; platelets may shield tumour cells to promote metastasis; and/or tumour-secreted cytokines may independently raise platelet counts.^19^ It is possible that all three of these processes can occur simultaneously in patients with thrombocytosis and underlying malignancy.

### Implications for research and practice

PPVs for cancer of 11.6% in males and 6.2% in females are very high, and well above the threshold value of 3% risk used for urgent investigation of suspected cancer in recent UK guidance,^2^ and even further above the 1% figure that patients would choose.^20^ This analysis shows that only two-thirds of patients had symptoms that would be recommended for urgent investigation in current NICE guidance; one-third had no relevant symptoms in the year before diagnosis, other than thrombocytosis. It is in this group that thrombocytosis has the greatest potential to prompt earlier diagnosis, where other symptoms have not yet developed. This strongly suggests that cancer should be considered when a result is received showing thrombocytosis, even if cancer was not initially suspected. The range of possible cancers is wide, so clinicians will probably have to seek additional features from the patient history and examination to select the most appropriate route for investigation. This analysis found that lung and colorectal cancers are more likely to be diagnosed in patients with thrombocytosis than in the general population, and breast and prostate cancers are less likely. The clinical situation differs from that of investigation for unprovoked thromboembolism, which remains controversial. This controversy follows a trial of alternative strategies for investigation of thromboembolism, with cancer yields of 3.2% in the limited testing group, and 3.9% in the extended testing group.^21^ The yield from investigation of thrombocytosis should be considerably higher.

To put these figures into context, the PPV of a breast lump in a female aged 50–59 years in primary care is 8.5% (95% CI = 6.7 to 11.0%).^10^ Moreover, the PPVs with thrombocytosis are higher than summary PPVs in recent meta-analyses of primary care studies of haemoptysis and lung cancer (3.5%, 95% CI = 1.6 to 7.5 in over-40s).^2^ The PPVs with thrombocytosis are similar to those of hypercalcaemia, which has a male cancer 1-year incidence of 11.5% and female cancer 1-year incidence of 4.1%.^22^

From a patient perspective, these results may be of even more value. Thrombocytosis has not generally been viewed as a risk marker for cancer, at least until recent national guidance included it for lung, oesophagogastric, and uterine cancers.^2^ The results of this study show that substantial proportions of lung and colorectal cancer diagnoses could be expedited by at least 2 months if thrombocytosis were to be routinely investigated. Based on findings from this study, even if only a conservative estimate of 5% of patients with cancer have thrombocytosis before diagnosis (16 500 out of 330 000 new cases annually in the UK), one-third of them have the potential to have their diagnosis expedited by at least 3 months; this equates to 5500 earlier diagnoses annually.

## Appendix 1. Symptoms reported by patients in the thrombocytosis and normal platelet count cohorts in the 28 days prior to their index date

**Table:**

**Symptom**	**Thrombocytosis (*n*= 31 261)**		**Normal platelet count (*n*= 7969)**	
	***n***	**%**	***n***	**%**
Cough	1221	3.9	151	1.9
Fatigue	942	3.0	205	2.6
Chest infection	935	3.0	63	0.8
Abdominal pain	826	2.6	133	1.7
Diarrhoea	716	2.3	63	0.8
Joint pain	704	2.3	149	1.9
Back pain	500	1.6	95	1.2
Chest pain	465	1.5	105	1.3
Shortness of breath	360	1.2	47	0.6
UTI	288	0.9	19	0.2
Dizziness	210	0.7	64	0.8
Palpitations	136	0.4	65	0.8

*UTI* = *urinary tract infections.*

## References

[1] Cancer Research UK Cancer mortality statistics.

[2] National Institute for Health and Care Excellence. (2015). Suspected cancer: recognition and referral. NG12..

[3] Neal RD, Tharmanathan P, France B (2015). Is increased time to diagnosis and treatment in symptomatic cancer associated with poorer outcomes? Systematic review. Br J Cancer.

[4] Shephard E, Neal R, Rose P (2013). Clinical features of kidney cancer in primary care: a case-control study using primary care records.. Br J Gen Pract.

[5] Hamilton W, Peters TJ, Round A, Sharp D (2005). What are the clinical features of lung cancer before the diagnosis is made? A population based case-control study. Thorax.

[6] Walker S, Hyde C, Hamilton W (2013). Risk of uterine cancer in symptomatic women in primary care: case-control study using electronic records.. Br J Gen Pract.

[7] Bailey SER, Ukoumunne OC, Shephard A, Hamilton W (2017). How useful is thrombocytosis in predicting an underlying cancer in primary care? A systematic review. Fam Pract.

[8] Hamilton W, Round A, Sharp D, Peters TJ (2005). Clinical features of colorectal cancer before diagnosis: a population-based case-control study. Br J Cancer.

[9] Stapley S, Peters TJ, Neal RD (2012). The risk of pancreatic cancer in symptomatic patients in primary care: a large case-control study using electronic records. Br J Cancer.

[10] Walker S, Hyde C, Hamilton W (2014). Risk of breast cancer in symptomatic women in primary care: a case-control study using electronic records.. Br J Gen Pract.

[11] Hamilton W, Peters TJ, Bankhead C, Sharp D (2009). Risk of ovarian cancer in women with symptoms in primary care: population based case-control study. BMJ.

[12] Shephard EA, Stapley S, Neal RD (2012). Clinical features of bladder cancer in primary care.. Br J Gen Pract.

[13] Stapley S, Peters TJ, Neal RD (2013). The risk of oesophago-gastric cancer in symptomatic patients in primary care: a large case-control study using electronic records. Br J Cancer.

[14] von Elm E, Altman DG, Egger M (2008). The Strengthening the Reporting of Observational Studies in Epidemiology (STROBE) statement: guidelines for reporting observational studies. J Clin Epidemiol.

[15] Dregan A, Moller H, Murray-Thomas T, Gulliford MC (2012). Validity of cancer diagnosis in a primary care database compared with linked cancer registrations in England. Population-based cohort study. Cancer Epidemiol.

[16] Boggon R, van Staa TP, Chapman M (2013). Cancer recording and mortality in the General Practice Research Database and linked cancer registries. Pharmacoepidemiol Drug Saf.

[17] Hamilton W, Lancashire R, Sharp D (2008). The importance of anaemia in diagnosing colorectal cancer: a case-control study using electronic primary care records. Br J Cancer.

[18] Pedersen LM, Milman N (1996). Prognostic significance of thrombocytosis in patients with primary lung cancer. Eur Respir J.

[19] Buergy D, Wenz F, Groden C, Brockmann M (2012). Tumor-platelet interaction in solid tumors. Int J Cancer.

[20] Banks J, Hollinghurst S, Bigwood L (2014). Preferences for cancer investigation: a vignette-based study of primary-care attendees. Lancet Oncol.

[21] Carrier M, Lazo-Langner A, Shivakumar S (2015). Screening for occult cancer in unprovoked venous thromboembolism. N Engl J Med.

[22] Hamilton F, Carroll R, Hamilton W, Salisbury C (2014). The risk of cancer in primary care patients with hypercalcaemia: a cohort study using electronic records. Br J Cancer.

